# Reconstructing the Genetic Potential of the Microbially-Mediated Nitrogen Cycle in a Salt Marsh Ecosystem

**DOI:** 10.3389/fmicb.2016.00902

**Published:** 2016-06-15

**Authors:** Francisco Dini-Andreote, Maria Julia de L. Brossi, Jan Dirk van Elsas, Joana F. Salles

**Affiliations:** Microbial Ecology Group, Genomics Research in Ecology and Evolution in Nature, Groningen Institute for Evolutionary Life Sciences, University of GroningenGroningen, Netherlands

**Keywords:** microbial succession, functional diversity, soil chronosequence, ecosystem functioning, metagenomics, qPCR

## Abstract

Coastal ecosystems are considered buffer zones for the discharge of land-derived nutrients without accounting for potential negative side effects. Hence, there is an urgent need to better understand the ecological assembly and dynamics of the microorganisms that are involved in nitrogen (N) cycling in such systems. Here, we employed two complementary methodological approaches (i.e., shotgun metagenomics and quantitative PCR) to examine the distribution and abundance of selected microbial genes involved in N transformations. We used soil samples collected along a well-established pristine salt marsh soil chronosequence that spans over a century of ecosystem development at the island of Schiermonnikoog, The Netherlands. Across the examined soil successional stages, the structure of the populations of genes involved in N cycling processes was strongly related to (shifts in the) soil nitrogen levels (i.e., ${\text{NO}}_{3}^{-}$, ${\text{NH}}_{4}^{+}$), salinity and pH (explaining 73.8% of the total variation, *R*^2^ = 0.71). Quantification of the genes used as proxies for N fixation, nitrification and denitrification revealed clear successional signatures that corroborated the taxonomic assignments obtained by metagenomics. Notably, we found strong evidence for niche partitioning, as revealed by the abundance and distribution of marker genes for nitrification (ammonia-oxidizing bacteria and archaea) and denitrification (nitrite reductase *nirK, nirS* and nitrous oxide reductase *nosZ* clades I and II). This was supported by a distinct correlation between these genes and soil physico-chemical properties, such as soil physical structure, pH, salinity, organic matter, total N, ${\text{NO}}_{3}^{-}$, ${\text{NH}}_{4}^{+}$ and ${\text{SO}}_{4}^{2-}$, across four seasonal samplings. Overall, this study sheds light on the successional trajectories of microbial N cycle genes along a naturally developing salt marsh ecosystem. The data obtained serve as a foundation to guide the formulation of ecological models that aim to effectively monitor and manage pristine and impacted salt marsh areas. Such models should account for the ecology as well as the historical contingency of N cycling communities.

## Introduction

Salt marshes rank among the most productive and valuable ecosystems in the world (Deegan et al., 2012; Bowen et al., 2013), yet they are sensitive and vulnerable to climate change and direct anthropogenic disturbances (Gedan et al., 2009). It is recognized that salt marshes have a major role in protecting coastal areas, for instance by removing land-derived compounds (Valiela and Cole, 2002; Sousa et al., 2008). Of particular importance, the runoff and groundwater discharges of agricultural fertilizers largely contribute to the influx of nutrients into these systems. In the case of nitrogen (N), these are either incorporated into plant biomass or removed by the local microbiota via nitrification and denitrification (Verhoeven et al., 2006). However, recent studies have indicated that such influxes of N forms can overwhelm the capacity of salt marshes to effectively remove N without deleterious effects to the ecosystem (Turner et al., 2009; Deegan et al., 2012). This occurs due to the increase in plant aboveground biomass that reduces the bank-stabilization of the roots. As a result, there is a progressive reduction of the geomorphic stability of the system, which leads to creek-bank collapse and salt marsh loss (Deegan et al., 2012).

In spite of their relevance, the current knowledge regarding the distribution of genes that govern nitrogen cycling in salt marshes and the effects of nitrogen input is still limited (Hamersley and Howes, 2005; Koop-Jakobsen and Giblin, 2010; Vieillard and Fulweiler, 2012; Kinney and Valiela, 2013). For instance, whereas the ammonia-oxidizing bacteria (AOB) were found to respond to N fertilization, ammonia-oxidizing archaea (AOA) remained unaffected (Peng et al., 2013). Moreover, no significant effect was found on the nitrogen-fixing and denitrifying bacterial communities when exposed to added N (Piceno and Lovell, 2000; Lovell et al., 2001; Bowen et al., 2011). Of particular importance, and lacking in these studies, is a thorough understanding of the distribution and drivers of N cycling communities in a naturally developing salt marsh ecosystem. Obtaining and inferring the genetic potential of these communities in such a system is critical, as it provides a baseline against which one can weigh the impact of N input with respect to community assemblage. Such a baseline will enhance our ability to manage the impacted areas at a landscape level.

A promising approach to assess the spatiotemporal patterns of N cycling communities relies on the use of chronosequences of soil formation. These model systems enable to study the dynamics of ecosystem development across multiple time scales (Walker et al., 2010), offering a setting to contrast and compare the patterns of community assembly across different successional stages. However, the study of spatiotemporal patterns of microbial communities in chronosequences is relatively recent, and it is under debate with respect to the drivers of community assembly and how these ultimately influence microbially-driven processes (Sigler and Zeyer, 2002; Nemergut et al., 2007; Brankatschk et al., 2011). As outlined by several authors, microbial communities exhibit successional trajectories that are tractable. In particular, recent studies have successfully disentangled the interplay between abiotic variables (edaphic factors) and the ecological mechanisms structuring the microbial communities across natural and disturbed soil chronosequences (Ferrenberg et al., 2013; Dini-Andreote et al., 2015).

In the present study, we investigated the N cycling microbial communities along a well-established chronosequence of soil formation. We specifically focused on the distributions and identities of organisms that are predicted to be involved in selected steps of the N cycle. A suite of complementary approaches was used to determine how the structure and functional capabilities of the target soil microbial communities shift over more than a century of natural ecosystem development. We thus analyzed soil sampled from five successional stages along replicated plots, representing distinctly vegetated sites under different abiotic conditions (i.e., tidal regime, salinity, pH and soil nutrients) (see Supplementary Figure 1 and Supplementary Table 1). The functional potentials of the communities were characterized by shotgun metagenomic profiling, whereas seasonal variations of specific N cycling genes were measured using quantitative PCR assays. We quantified the genes encoding (1) subunits of enzymes involved in N fixation (nitrogenase reductase, *nifH*), (2) nitrification (ammonia monooxygenase, *amoA*, of both AOB and AOA) and (3) denitrification (nitrite reductase *nirS* and *nirK* and nitrous oxide reductase *nosZ* from clades I and II). The whole dataset was used to test the overarching hypothesis that the genes involved in the different N transformations change not only in abundance but also with respect to the identity of the taxonomic groups along the ecological succession. We also examined whether the shifts are related to important abiotic variables, such as primary net productivity (Brankatschk et al., 2011) and soil nutrient status, i.e., levels of carbon (Liu and Greaver, 2010) and nitrogen (LeBauer and Treseder, 2008).

## Materials and methods

### Sampling location and data collection

Soil samples were collected along a well-established salt marsh chronosequence located at the island of Schiermonnikoog (N53°30″ E6°10″), The Netherlands (Supplementary Figure 1). This chronosequence is formed through the constant deposition of silt and clay particles (carried by sea currents and winds directed west-to-east) that accumulate on top of the underlying sandflats, causing the island to progressive extend eastwards (Olff et al., 1997; Schrama et al., 2012). Salt marsh age at each stage of the succession was estimated from topographic maps, aerial photographs and the thickness of the sediment layer accumulating on top of the underlying sand layer. In addition, permanent plots have monitored the space-for-time replacement in this system for more than 20 years (Van Wijnen et al., 1997). For this study, five different soil successional stages were identified and estimated as 0, 5, 35, 65 and 105 years of soil development in 2012 (the sampling year) (referred in the main text and figures as “stages 0, 5, 35, 65, 105”). Soil samples were collected in May, July, September and November 2012. Triplicate plots (5 × 5 m^2^) were established at each identified soil successional stage (separated 25 m from each other) at the same base of elevation—position at the initial elevation gradient on the bare sand flats with a base elevation of 1.16 m ± 2.2 cm (mean ± SE) above Dutch ordinance level. Importantly, differences in the base elevation reflect differences in inundation regimes, therefore having strong influences on the dynamics and the fate of succession (Olff et al., 1997). Soil samples were collected in each plot by randomly taking 20 soil cores (5 cm diameter, 10 cm depth), using aseptic techniques, to represent a composite sample. Samples were placed in a sterile plastic bag, sealed and transported to the laboratory (< 24 h). All samples were sieved (4 mm mesh size) under sterile conditions and stored at −20°C for total DNA extraction and at 4°C for physico-chemical measurements. For each sample, we quantified the soil physical structure (silt:clay:sand %) and chemical content of total organic matter (SOM), nitrate (N-${\text{NO}}_{3}^{-}$), ammonium (N-${\text{NH}}_{4}^{+}$), sulfate (S-${\text{SO}}_{4}^{2-}$), sodium (Na) and pH. For detailed information on the soil physico-chemical analyses see Dini-Andreote et al. (2014). For soil physico-chemical properties see Supplementary Table 1. Total soil DNA was extracted from 0.5 g of soil using the MoBio PowerSoil DNA isolation kit (MoBio Laboratories, Carlsbad, CA, USA). Extracted DNA samples were quantified using the Quant-iT PicoGreen dsDNA assay kit (Invitrogen, Carlsbad, CA, USA) on a TECAN infinite M200 Pro (Maennedorf, Switzerland) plate reader reading at 485 nm excitation and 530 nm emission. All samples were standardized at equal concentrations for further analysis.

### Shotgun metagenomics

Shotgun metagenomic sequencing was conducted following the procedure described in the Illumina TrueSeq DNA sample preparation protocol. The triplicated samples for each successional stage collected in July 2012 were subjected to shotgun metagenomic profiling (*n* = 15). Seasonal variations in the abundance of marker genes were later interrogated by primer-specific quantitative PCR assays (see below). In brief, aliquots of each DNA sample were mechanically sheared before entering the Illumina library generation protocol. Libraries were size-selected to 170–180 bp using an agarose gel. Sequencing was carried out in a paired-end (2 × 100 bp) Illumina HiSeq2000 run at the Argonne National Laboratory in the Next Generation Sequencing Core (NGS). Raw, unassembled Illumina reads were paired, dereplicated and quality filtered in MG-RAST (Meyer et al., 2008). Putative open reading frames on the quality-controlled sequences were called using FragGeneScan (Rho et al., 2010). Metagenomes were functionally annotated using BLASTX searches against the KEGG (Kyoto Encyclopedia of Genes and Genomes) Orthology (KO) identifiers (Kanehisa et al., 2008). For the taxonomic assignments of selected KOs, sequences of specific KOs were retrieved and annotated against the M5nr (an MD5 nonredundant database) (Wilke et al., 2012) using the best-hit organismal classification method. Functional and taxonomic annotations of sequences were carried out with a maximum *e*-value cutoff of 10^−5^, a minimum percent identity cutoff of 60% and a minimum alignment length cutoff of 15. All sequence data have been deposited in the MG-RAST database. The reference IDs of the metagenomes are provided in Supplementary Table 2.

### Quantitative PCR analysis

Functional marker genes encoding subunits of enzymes involved in N fixation (*nifH*), nitrification [*amoA* of ammonia-oxidizing bacteria (AOB) and archaea (AOA)] and denitrification (*nirS, nirK* and *nosZ* clades I and II), in addition to the phylogenetic marker 16S rRNA gene for bacteria and archaea, were quantified using quantitative PCR (qPCR) assays run on a ABI Prism 7300 Cycler (Applied Biosystems). For the qPCR assays, all collected samples were considered, including seasonal time points (May, July, September and November) (*n* = 60). We applied Sybr Green-based quantification assays using the Power SYBR Green PCR Master Mix (Applied Biosystems, Frankfurt, Germany). Reaction volumes were 25 μL containing one-fold PCR master mix. PCR conditions, efficiencies, primers and calibration standards used are shown in Table 1. The specificity of the amplification products was confirmed by melting curve analyses, and the expected sizes of the amplified fragments were checked in a 1.5% agarose gel. Two independent quantitative PCR assays were performed for each gene and four no-template controls were run for each qPCR assay, which resulted in null or negligible values. Standard curves were generated over five orders of magnitude, i.e. from 10^3^ to 10^8^ copies of template, using a plasmid containing specific marker genes (Table 1). The qPCR efficiency (*E*) was calculated according to the equation *E* = [10^(−1∕slope)^−1]. Possible inhibitory effects were checked by spiking samples with a range of known concentrations of the plasmid. No apparent inhibition was observed for any of the quantified genes. Data were first calculated as log copy numbers per gram of dry-weight soil (Supplementary Figure 2). Since the sizes of the bacterial and archaeal communities change significantly over the successional gradient (see Supplementary Figure 2), our data are shown as the ratio between the abundance of each N cycling gene and its respective organismal abundance (either bacteria or archaea), as a percentage.

**Table 1:** **Quantitative PCR reaction composition, thermal cycling, source of calibration standards and primer references used in this study**.

**Target gene**	**Reaction conditions**							**Source of calibration (standard curve)**	**Primer name and reference**
	**F- and R-primer (pmol μl^−1^)**	**BSA (μg μl^−1^)**	**DMSO (μg μl^−1^)**	**Denaturation time at 95°C (s)**	**Annealing time and temperature**	**Elongation time at 72°C (s)**	**qPCR efficiency (%)**
*nifH*	0.26	0.6	0	60	27 s at 55°C	60	94	*Bradyrhizobium liaoningense*	FPGH19/ PolR (Simonet et al., 1991; Poly et al., 2001)
*amoA* (AOA)	0.7	0.6	0	40	30 s at 56°C	60	98	Soil clone	Arch amoA-1F/Arch amoA-2R (Francis et al., 2005)
*amoA* (AOB)	0.2	0.6	0	45	45 s at 60°C	45	100	Soil clone	amoA 1F/amoA 2R (Rotthauwe et al., 1997)
*nirK*	0.3	0.6	30	15	30 s at 58°C	30	99	*Pseudomonas fluorescens*	nirK876/nirK5R (Braker et al., 1998; Henry et al., 2004)
*nirS*	0.3	0.6	30	30	60 s at 57°C	45	98	*Pseudomonas aeruginosa*	nirS cd3af/nirS R3cd (Michotey et al., 2000; Throback et al., 2004)
*nosZ clade I*	1	0.6	0	15	30 s at 60°C	30	102	*Pseudomonas aeruginosa*	nosZ2F/nosZ2R (Henry et al., 2006)
*nosZ clade II*	2	0.6	0	30	30 s at 54°C	45	107	*Gemmatimonas aurantiaca*	nosZ-II-F/nosZ-II-R (Jones et al., 2013)
*Total bacteria*	0.8	0.4	0	27	60 s at 62°C	30	106	*Serratia plymuthica*	FP16S/RP16S (Bach et al., 2002)
*Total archaea*	0.3	0.4	0	20	30 s at 60°C	27	104	Soil clone	Arch-967F/Arch-1060R (Cadillo-Quiroz et al., 2006)

*AOB, ammonia-oxidizing bacteria; AOA ammonia-oxidizing archaea*.

### Statistical analyses

Cross-soil comparisons were made using Bray-Curtis similarities calculated based on normalized and square-root transformed count matrices of unique KOs. As the size of the metagenomic libraries varied stochastically by soil sample (Supplementary Table 2), raw counts were normalized to metagenomic library size to account for inconsistent sample depth. This facilitated comparison between soils prior to Bray-Curtis calculations. Principal coordinate analyses (PCO) and PERMANOVA (Anderson, 2001) were performed using the homonymous routines in PRIMER6+ (Clarke and Gorley, 2006) for the complete functional community profiling data (KO annotations) and a selection of KOs specifically involved in N cycle transformations (i.e., Nitrogen metabolism [PATH:ko00910]). Significance levels calculated in PERMANOVA were determined with 10^3^ permutations. All measured soil physico-chemical properties were checked for normality using the Shapiro-Wilk test and further log(*x*+1) transformed—with the exception of pH—to improve normality and homoscedasticity for multivariate statistical analyses. The correlation strength and significance between the structure of the N cycling communities and the soil metadata were determined using RELATE (a nonparametric Mantel type test), run with 10^3^ permutations. We further used a nonparametric multivariate regression between normalized soil physico-chemical parameters and the Bray-Curtis similarity matrix of N cycling communities implemented as distance-based linear modeling (DistLM) (McArdle and Anderson, 2001), run with 10^3^ permutations. The model was built using a step-wise selection procedure and the adjusted R^2^ selection criterion. We further used BEST to select for the best combination of less-colinear factors—previously selected by Marginal DistLM—to be incorporated in the models using the “forward” procedure within DistLM. The selected soil parameters were subsequently used to build a constrained ordination plot using the best-fitted model in a distance-based redundancy analysis (dbRDA) (Legendre and Anderson, 1999). These analyses were conducted using PRIMER6 and PERMANOVA+ (PrimerE Ltd, Ivybridge, UK).

For this study, the functional metagenomic analyses focused on the genetic potential of communities involved in N transformations. Marker genes (KOs) were selected as previously reported (Lauro et al., 2011; Llorens-Marès et al., 2015) (see Supplementary Table 3 and **Figure 2**, for a full description). Predicted KOs that segregated significantly between successional stages were identified using random forest analysis (Breiman, 2001) with 1,000 trees followed by the Boruta algorithm for feature selection (average *z*-scores of 1,000 runs > 4) (Kursa and Rudnicki, 2010). These analyses were carried out in R using the packages RandomForest v4.6-7 and Boruta v3.0. Heatmaps were constructed based on *z*-score transformed functional annotations to improve normality and homogeneity of the variances. The genetic potentials to perform specific steps in the N biogeochemical cycle of the salt marsh soils were assessed using the combination of these selected marker genes (Lauro et al., 2011; Llorens-Marès et al., 2015), where each gene combination is averaged (multiple enzymes/subunits in the same conversion step) or summed (multiple pathways performing the same conversion step). For a detailed list of selected KOs involved in each step of the N cycle, see Supplementary Table 3.

## Results

### Structure of N cycling communities in a developing salt marsh chronosequence

**Figure 1:**
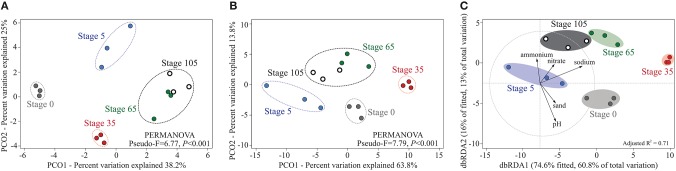
**Structure of the microbial communities along the successional gradient as determined by shotgun metagenomics**. Plots illustrating distances between microbial communities in individual samples. **(A)** PCO based on complete functional community profiles (KO annotations). **(B)** PCO based on selected KOs involved in the N cycle (i.e., Nitrogen metabolism [PATH:ko00910]). Significant clusters are indicated by dashed lines (PERMANOVA, *P* < 0.05; see Supplementary Table 4 for details). **(C)** Distance-based redundancy analysis (dbRDA) illustrating the “best” fitting DistLM model (adjusted *R*^2^ = 0.71) containing forward selected predictor variables. Axis legends include % of variation explained by the fitted model and % of total variation explained by the axis.

We analyzed the shotgun metagenomic data from the soil samples taken from triplicate plots along five successional stages (sampling time: July; *n* = 15) in the Schiermonnikoog chronosequence. The sequenced soil metagenomes averaged 1.9 ± 0.7 (mean ± SD) gigabases, with average read lengths of 165 bp. Of the total read counts in each metagenome, 12.6 ± 0.9% could be assigned to KOs and thus to specific functions (*e*-value 10^−5^) (see Supplementary Table 2 for details). Principal coordinate analysis (PCO) of the annotated KOs revealed a clear separation of functional community structures when compared across different soil successional stages (Figure 1A) (PERMANOVA, Pseudo-F = 6.77, *P* < 0.001; for pairwise PERMANOVAs between consecutive soil stages, see Supplementary Table 4). From the annotated KOs, we then selected a set of marker genes that report on specific N cycle transformations (i.e., nitrogen metabolism [PATH:ko00910]). Differences in functional community structures occurred in a similar manner when only the KOs involved in N cycling were considered (Figure 1B) (PERMANOVA, Pseudo-F = 7.79, *P* < 0.001; see also Supplementary Table 4).

The investigated soil successional stages had distinct edaphic properties, as previously described in detail (Dini-Andreote et al., 2014, 2015, 2016; see Supplementary Table 4). We thus examined a possible coupling of the N cycling data with the soil metadata. First, the functional community structures were weakly but significantly correlated with soil physico-chemical properties (RELATE ρ = 0.249, *P* = 0.013). Analyses of the individual soil parameters by Marginal DistLM resulted in the assertion that (levels of) sodium (Pseudo-F = 3.66, Proportion = 0.22), nitrate (Pseudo-F = 2.37, Proportion = 0.15), ammonium (Pseudo-F = 2.31, Proportion = 0.15), pH (Pseudo-F = 1.95, Proportion = 0.13) and sand content (Pseudo-F = 1.58, Proportion = 0.10) were the best predictors of the structures of the N cycling communities. Collectively, these selected parameters correlated marginally with the N cycling community structures (BEST ρ = 0.262, *P* = 0.049). The best-fitted DistLM model, using the “forward” procedure of selected predictor variables, as shown by dbRDA, explained 73.8% of the total variation with an adjusted R^2^ of 0.71 (Figure 1C).

### Genetic potentials and taxonomic assignment of N cycling communities in salt marsh soils

**Figure 2:**
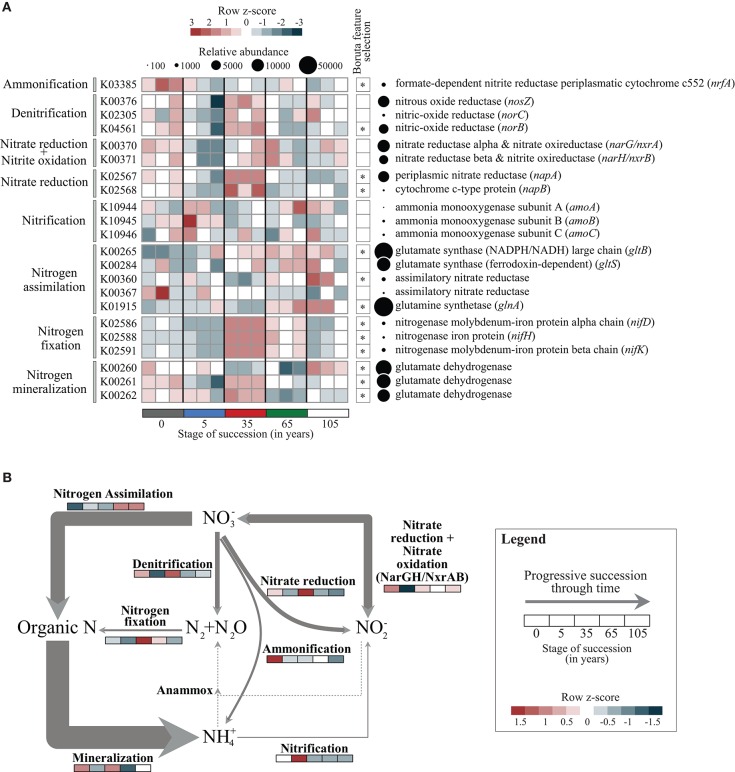
**Distribution of genes involved in the N cycle in salt marsh soils. (A)** Distribution of KOs involved in N cycle transformations in samples collected along the salt marsh soil chronosequence. The heatmap displays the relative abundance (row *z*-scores) of KOs across all samples (triplicate plots per stage of succession). KOs that differentially segregated across soil successional stages were identified by random forest analysis with Boruta feature selection (average *z*-scores of 1000 runs > 4) (see Supplementary Table 6). Circles are proportional to the relative abundance of each gene family in all samples. **(B)** Genetic potential for different steps of the N cycle in salt marsh soils using a combination of normalized marker genes (see Materials and Methods and Supplementary Table 3 for details). Arrow sizes are proportional to the genetic potential of the nitrogen transformation (100%, see Supplementary Tables 3, 7 for details). Differences across successional stages of each step are shown by *z*-score heatmap lines indicated in each N transformation.

We used the relative abundances of selected marker genes obtained by metagenomics as proxies for their potential relevance (i.e., genetic potential) in different steps of the N cycle. We start by depicting the normalized relative abundance of selected marker genes involved in N transformations across the different successional stages. The data showed that genes related to N assimilation and mineralization encompassed the major proportion of genes present across all successional stages (61.8 ± 3.4% and 22.3 ± 1.3%, respectively) (Supplementary Table 5, Figure 2A). Three genes encoding glutamate dehydrogenase (involved in N mineralization; K00260, K00261, K00262) were found to have a differential distribution along the succession (Boruta feature selection average *z*-score of 1,000 runs > 4). The taxa involved in N mineralization were affiliated with heterotrophic free-living organisms at early successional stages, mainly Actinomycetales (7.8%) and Burkholderiales (7.6%), whereas at stage 105 the Actinomycetales (10.6%) were followed by Rhizobiales (7.9%), encompassing the genera *Bradyrhizobium, Methylobacterium, Rhizobium* and *Xanthobacter*. Here, Burkholderiales encompassed only 4.5% of the total gene sequences. Next, the potential for nitrogen fixation (*nif* genes) was observed in all soil sites, significantly segregating across these and peaking at stage 35 (2.4%) followed by the late successional sites (stages 65 and 105, 1.4 and 0.6%, respectively). The taxonomic assignment of the *nif* genes was partitioned mostly among Desulfuromonadales (23.5%) and Desulfovibrionales (21.3%) at the initial soil stage. However, as succession proceeds, the relative contribution of N-fixing Desulfuromonadales increased, reaching 31.8% at stage 35 and peaking at the soil stages 65 and 105 (43.9 and 42.1%, respectively). Genes involved in nitrification were present at low abundances in all soil sites (0.18 + 0.07%), followed by ammonification (0.7 + 0.19%). The levels of the latter genes peaked at stage 0 (1.02%), with the underlying taxa being mostly affiliated to the orders Myxococcales (29.4%) (genera *Anaeromyxobacter, Myxococcus, Sorangium*) and Desulfuromonadales (16.9%) (genera *Geobacter* and *Pelobacter*). Genes involved in denitrification (5.2 + 0.8%) were found across all successional stages. They peaked in intermediate site (stage 35) at 6.22%, which was followed by stage 0 at 5.8% and stage 105 at 5.0%. The main denitrifying taxa were Flavobacteriales (17.2%) and Cytophagales (7.4%) at stage 0. At stage 35, Flavobacteriales (15.6%), Bacteroidetes Order II Incertae sedis belonging to the family Rhodothermaceae (genera *Rhodothermus* and *Salinibacter*) (5.5%) were prominent, whereas Flavobacteriales (19.8%) and Rhizobiales (5.5%) prevailed at stage 105. We also found significant differences in the distribution and abundances of genes encoding enzymes involved in nitrate reduction (*napA*, periplasmic nitrate reductase; *napB*, cytochrome c-type protein). These genes peaked at stage 35 (4.4%), with the underlying taxa being mostly affiliated to Alteromonadales (10.9%), Burkholderiales (8.1%), Campylobacterales (9.2%) and Puniceicoccales (7.7%). We further examined the genetic potential of genes involved in N cycle transformations in salt marsh soils using a previously described approach (for details see Lauro et al., 2011; Llorens-Marès et al., 2015; Supplementary Table 3 and Figure 2B).

### Quantitative assessment and seasonal variation of N cycling gene abundances

**Figure 3:**
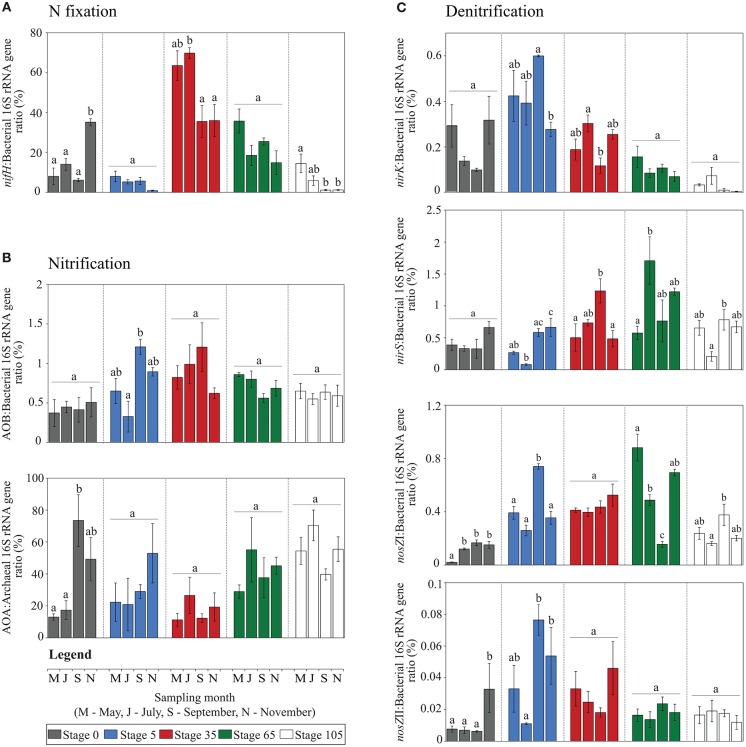
**Relative abundances of N cycling genes in five successional stages of the salt marsh chronosequence**. Data encompass four sampling times (May, July, September and November 2012). Values are shown as the ratio between the abundance of each N cycling gene and the respective organismal abundance (either bacteria or archaea), in percentage. **(A)** N fixation (*nifH* gene), **(B)** Nitrification [*amoA* gene of ammonia-oxidizing bacteria (AOB) and archaea (AOA)] and **(C)** denitrification (*nirS, nirK* and *nosZ* clades I and II genes). Bars represent average values ± standard error (SE) (*n* = 3) and letters above each bar describe seasonal variations within each stage of succession (ANOVA with Tukey's *post-hoc* test, *P* < 0.05).

Quantitative PCR (qPCR) was used to examine the abundances of nitrogen fixers (*nifH*), ammonia-oxidizing bacteria (AOB-*amoA*), ammonia-oxidizing archaea (AOA-*amoA*) and denitrifiers (*nirK, nirS* and *nosZ* clades I and II). In order to address potential within-stage seasonal variations in the N cycling marker gene abundances, quantifications were performed in soil samples collected at five successional stages (triplicate plots per stage) along the soil chronosequence, at four sampling times (samples collected in May, July, September and November 2012) (*n* = 60). Importantly, the population sizes of bacteria and archaea (as estimated by the copy numbers of 16S rRNA genes) varied over 10- to 100-fold across all stages of the chronosequence and seasonal variations were observed in some cases (Supplementary Figure 2). Given such variation, our data are shown as the ratio between the abundance of each N cycling gene and its respective organismal abundance (either bacteria or archaea), in percentage (termed “relative abundance”) (Figure 3). For absolute quantifications of each individual gene, shown as log copy numbers per gram of dry-weight soil, see Supplementary Figure 2.

#### Nitrogen fixation (bacterial *nifH* gene)

**Figure 4:**
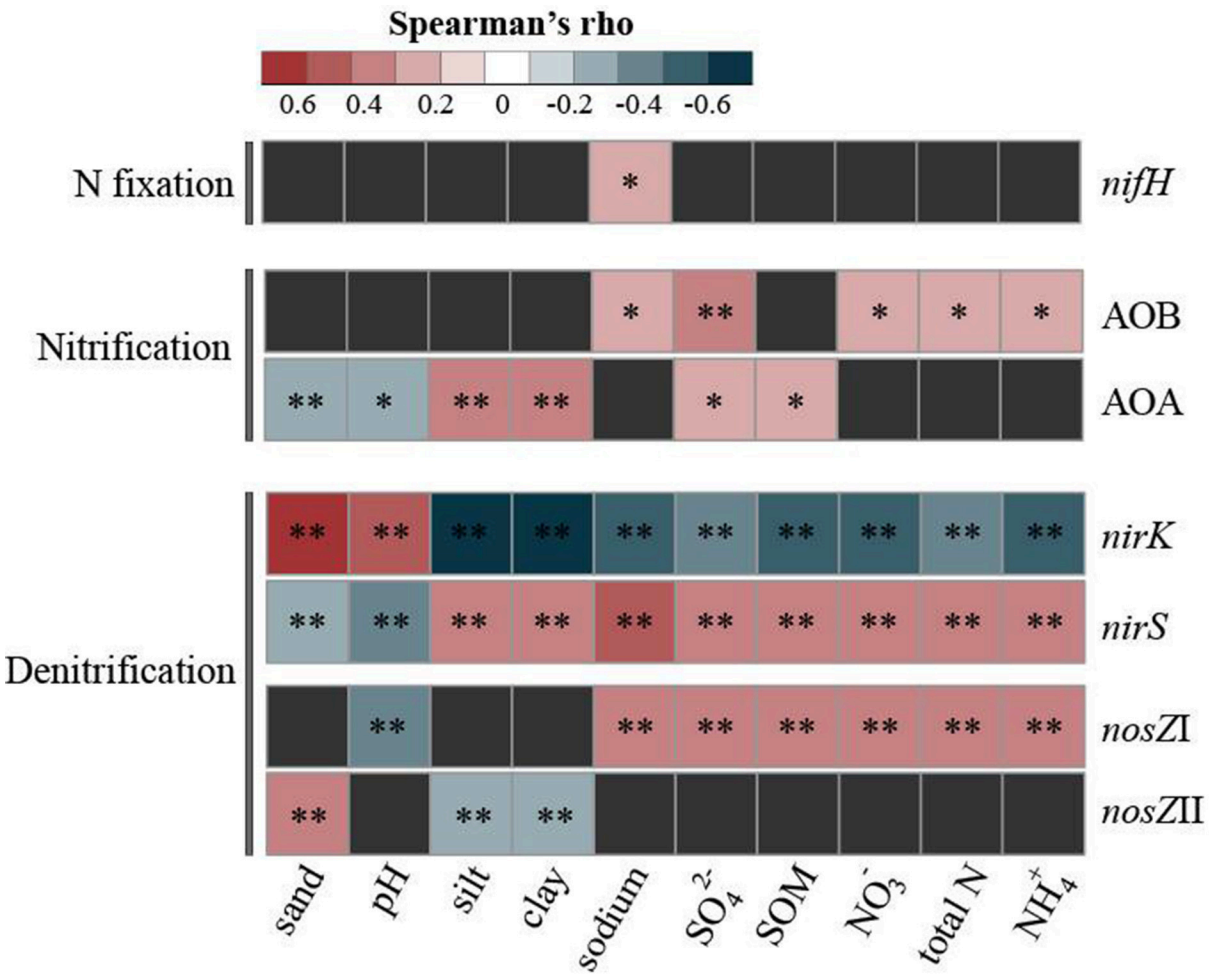
**Correlational analyses between the relative abundance of each N cycling gene and the soil physico-chemical properties**. The heatmap displays significant positive (ρ > 0) and negative (ρ < 0) Spearman's correlations. ^*^P < 0.05 and ^**^P < 0.01.

The relative abundance of the *nifH* gene peaked at the intermediate site (stage 35, seasonal variation 51 ± 18%, average ± SD), followed by stage 65 (23 ± 9%), being lowest at stages 5 (5 ± 3%) and 105 (4–6%) and slightly higher at stage 0 (16 ± 13%). Interestingly, these results corroborate the quantifications of this gene obtained by comparative metagenomics (see Figure 2). Seasonal variations in the N fixer population sizes were observed for stages 0, 35 and 105 (ANOVA with Tukey's *post-hoc* test, *P* < 0.05) (see Figure 3 for details). We also found the relative abundance of the *nifH* gene to weakly but significantly correlate (ρ = 0.27, *P* < 0.05) with variations in sodium concentration across the samples (Figure 4).

#### Ammonia oxidation (bacterial AOB-*amoA* and archaeal AOA-*amoA*)

The abundance of the ammonia monooxygenase gene was quantified for both AOB and AOA. Independent of the stage of succession, the abundance of AOB was always higher than that of AOA (AOB:AOA ratios of >1). Moreover, the abundance of total bacteria was higher than that of archaea by ca. 1,000-fold (Supplementary Figure 2). Normalization of *amoA* gene numbers as relative abundance in the respective organismal groups indicated that the AOB accounted for a small proportion of the total bacterial communities (ca. 0.5–1.5%), being higher at stage 35 and displaying seasonal variations at stage 5 (Figure 3B). Conversely, the relative abundance of the *amoA* gene in the archaeal communities ranged from ca. 16 to 77%, displaying a slightly opposite pattern, i.e., being proportionally lowered at the intermediate stage (stage 35) and displaying only significant seasonal variations at the initial soil site (stage 0) (Figure 3B). These differences were also evidenced by the distinct correlations of the patterns with the soil metadata. Whereas AOB correlated with the soil chemical parameters (i.e., sulfate, sodium, total N, ${\text{NH}}_{4}^{+}$ and ${\text{NO}}_{3}^{-}$ contents; ρ-values ranging from 0.22 to 0.34, *P* < 0.05), AOA correlated with soil physical structure (i.e., silt and clay content, ρ-values ca. 0.33, *P* < 0.01; sand content, ρ = −0.32, *P* < 0.01). For the latter, only marginal correlations were found with SOM (ρ = 0.22, *P* < 0.05), sulfate concentrations (ρ = 0.28, *P* < 0.05) and soil pH (ρ = −0.3, *P* < 0.05) (Figure 4).

#### Denitrification (bacterial *nirS, nirK* and *nosZ* clade I and clade II)

To quantify denitrifying bacteria, genes encoding nitrite reductase (*nirK* and *nirS*) and nitrous oxide reductase (*nosZ* clade I and II) were used. The relative abundances of the *nirK* and *nirS* genes were clearly influenced by the conditions prevailing at the local sites. This was evidenced by the opposing relation of their respective distributions with the soil metadata. In brief, whereas *nirK* was positively and significantly correlated with parameters prevailing at initial soil stages (higher pH and sand content) (ρ = 0.70 and 0.69, respectively; *P* < 0.01), *nirS* correlated with conditions prevailing at late successional stages (higher nutrient contents—SOM, total N, ${\text{NH}}_{4}^{+}$ and ${\text{NO}}_{3}^{-}$—${\text{SO}}_{4}^{2-}$ and sodium concentrations, and silt and clay contents) (ρ-values ranging from 0.32 to 0.55, *P* < 0.01) (Figure 4). The relative abundances of these genes also revealed opposite trends along the successional gradient: *nirK* peaked at the initial soil sites, ranging from ca. 0.21 ± 0.14 to 0.42 ± 0.16% (average ± SD) at stages 0 and 5, respectively; whereas *nirS* was highest at the intermediate and late sites, ranging from ca. 0.58 ± 0.28 to 1.06 ± 0.6% at stage 105 and 65, respectively (Figure 3). For the seasonal within-stage variations of these genes, see Figure 3. We also quantified the abundance of the nitrous oxide reductase gene (*nosZ*), which has been recently shown to occur across two distinct phylogenetic clades (see Discussion for details). Across all successional stages, we found *nosZ* clade I to occur at ca. 10-fold higher abundances than clade II (Figure 3C). The relative abundances of these clades did not display a clear inversely distributed pattern along the chronosequence. However, they clearly correlated with different soil parameters. For instance, whereas *nosZ* clade I correlated with (shifts in) soil chemical properties (i.e., sodium, SOM, sulfate and N—total, nitrate and ammonium) (ρ-values ranging from 0.32 to 0.4, *P* < 0.01) (pH, ρ = −0.35, *P* < 0.01), *nos*Z clade II correlated only with variations in soil physical structure: sand (ρ = 0.32, *P* < 0.01), silt (ρ = −0.32, *P* < 0.01) and clay (ρ = −0.33, *P* < 0.01). Both *nosZ* clades also displayed different within-stage seasonal variations along the chronosequence, providing additional support for their niche partitioning (Figure 3C).

## Discussion

The increasing discharges of agricultural fertilizers into coastal ecosystems have been shown to cause a progressive transition of creek-edge and bay-edge marshes into mudflats and wider creeks worldwide (MacGarvin, 2001; Tiner et al., 2006; Deegan et al., 2012). Thus, to prevent such ecological distress, it is crucial to examine the capacity of salt marshes to cycle N and to deal with exogenous N influxes (e.g., Lovell et al., 2001; Bowen et al., 2011; Peng et al., 2013). In spite of the urgency, the current knowledge of how microbial N cycling populations are ecologically assembled in salt marshes is still rudimentary. Here, we examined the successional signatures of microbial N-cycling genes across a naturally developing salt marsh chronosequence. Previous studies have made use of these soil sites to examine the patterns and mechanisms driving bacterial and fungal community assembly and dynamics at the phylogenetic level (see Dini-Andreote et al., 2014, 2015, 2016). In order to build on this knowledge, we here examined the distribution of genes predicted to encode nitrogen cycling enzymes in this system. Our results highlight whether or not the observed shifts relate to important abiotic variables. Last, we provided evidence that metabolically redundant genes involved in the nitrification and denitrification pathways are niche partitioned.

### Nitrogen input at the initial stages of the soil chronosequence

Emerging terrestrial systems are often characterized by low nutrient content and scarce vegetation (Sigler and Zeyer, 2002; Brankatschk et al., 2011). In the Schiermonnikoog chronosequence, the initial soil sites (stages 0 and 5) had less than 20% vegetation cover, whereas the intermediate (stage 35) and late sites (stages 65 and 105) were densely vegetated (Schrama et al., 2012). Thus, the initial input of organic materials by root exudates and plant litter was presumably low. Hence, N fixation, as well as autotrophic CO_2_ incorporation, at early successional sites might be critical for primary production (Kohls et al., 1994). Interestingly, using metagenomics and direct qPCR, our data revealed a low genetic potential for N fixation and low *nifH* gene copy numbers at stages 0 and 5 (Figures 2B, 3A). This finding corroborates those of Brankatschk et al. (2011) and Nemergut et al. (2007), who also found that the lowest N fixation rates occurred in the bulk soil of the initial, rather than later, stages of soil development at the forefields of the Damma glacier in Switzerland and the Puca glacier in Peru. Moreover, we did not find a systematic shift in the taxonomic distribution of organisms holding N fixation genes. For instance, there was a clear (relative) dominance of anaerobic sulfate reducers as N fixers throughout the current study, as organisms belonging to the Desulfuromonadales increased progressively along the successional gradient (see Results for details). In addition, whereas free-living autotrophic N-fixers such as cyanobacteria were expected to abound in the early stages (Bolhuis and Stal, 2011; Fan et al., 2015), anaerobic N fixers, specifically belonging to the Desulfuromonadales and Desulfovibrionales, prevailed. This finding potentially relates to our sampling strategy, which focused on the “bulk soil” part of the initial stages, whereas the referenced studies focused on microbial mats.

In contrast to the (relatively) low genetic potential for N fixation, uptake of organic nitrogen compounds that came about as a result of mineralization seems to be the major N cycling step at the early stages of this ecosystem development. This is supported by the high abundance of glutamate dehydrogenase genes, which are proxies for mineralization. These genes were mostly affiliated with heterotrophic free-living organisms like Actinomycetales and Burkholderiales at the early stages, but changed systematically as succession proceeded (see Results for details). In line with these arguments, it is worth mentioning that (marine-derived) C substrates are constantly being provided for mineralization at these initial soil sites (Schrama et al., 2012). These external amendments of organic materials occur mostly through the adjective effect of the tides (i.e., daily cycles of inundation and water retraction). This process has not only been shown to carry high loads of marine-derived nutrients toward the coast (Schrama et al., 2012), but it may also have been the key cause of the largely stochastic assembly of the microbial communities at the early stages of this chronosequence (Dini-Andreote et al., 2015).

### Evidence for niche partitioning in nitrifying and denitrifying communities

Nitrification in soils is often rate-limited by the first step, the oxidation of ammonia, which is driven by AOB and AOA. Across all successional stages, the abundance of the genes involved in nitrification was very low in the metagenomes, yet they were detected at a higher resolution in the qPCR assays. Interestingly, the abundance distributions of nitrification genes across successional stages displayed opposite patterns, providing an indication that niche partitioning influences the abundances of *amoA* from AOB and *amoA* from AOA across the chronosequence (Figure 3B). This finding feeds the current debate on the drivers of the AOB and AOA in soils (e.g., Sterngren et al., 2015). Mounting evidence supports a clear niche partitioning between these organismal groups. For instance, the physiological characterization of culturable ammonia oxidizers has pointed toward their different tolerances to ammonium concentrations—whereas AOA isolates are inhibited at 2–20 nM (Tourna et al., 2011; Hatzenpichler, 2012), AOB tolerate a concentration of up to 50–1000 nM (Koops and Pommerening-Röser, 2006). In our study, AOB always outnumbered AOA (AOB:AOA ratios of >1), which is consistent with other data from estuarine and coastal studies (e.g., Santoro et al., 2008; Caffrey et al., 2010; Wankel et al., 2011). Further support for niche partitioning was found in the significant correlations between AOB abundance and sulfate concentration (Spearman, ρ = 0.34, *P* < 0.01), and the marginal positive correlations with other soil chemical parameters (see Figure 4 for details). As for the AOA abundances, they correlated mostly with soil physical structure (i.e., sand:silt:clay content). Collectively, our data are consistent with a suite of other studies that indicate, in order of importance, that dissolved oxygen, temperature (directly affected by the soil structure), soil salinity, sulfate and N availability exert significant influences on the abundance and composition of the ammonia-oxidizing communities in soils (e.g., Francis et al., 2003; Ward et al., 2007; Santoro et al., 2008; Moin et al., 2009).

Denitrification, a facultative respiratory pathway in which nitrate is reduced to nitrogen gas through the intermediates nitrite, nitric oxide and nitrous oxide, is a wide-spread process carried out by many bacterial and archaeal taxa (Jones and Hallin, 2010). Here, we initially focused on the abundance of genes for two functionally equivalent, yet structurally distinct, nitrate reductase encoding the reduction of nitrite to nitric oxide (*nirK* and *nirS*). We found a clear separation of the *nirS* and *nirK* types based on the habitat categories studied, which supports the idea that habitat selective factors exert a differential effect on organisms with different *nir* types (Hallin et al., 2009). Despite the overrepresentation of *nirK* over *nirS* across all successional stages (*nirK:nirS* ratios of >1 at the level of gene copies per gram of soil), we found their relative abundances to vary in a distinct manner along the succession. That is, *nirK* peaked at the initial soil sites (stages 0 and 5), and steadily decreased over time, whereas *nirS* showed the opposite pattern (Figure 3C). These findings are in striking conflict with the idea that *nirS* types dominate in marine habitats whereas *nirK* prevails in terrestrial systems (Jones and Hallin, 2010). Salinity has been suggested as an effective modulator of *nir* types (Jones and Hallin, 2010), and one explanation for this discrepancy might be the progressive accumulation of sodium (Na) in later stages of the chronosequence. Specifically, the initial soil sites had relatively low levels of Na (ca. 1.8–2.4% by weight) as compared with the intermediate and late successional stages (ca. 13.8–14.4%) (Dini-Andreote et al., 2014, 2015; Supplementary Table 1). The opposing correlational patterns of *nir* types and the soil parameters (including Na) (Figure 4) supports the idea of niche partitioning. This finding is also consistent with Smith and Ogram (2008) suggesting that denitrifying communities harboring different *nir* types respond differently to environmental gradients. Moreover, we also revealed the habitat preferences of two phylogenetic clades of nitrous oxide reductase (nitrous oxide conversion to dinitrogen; gene *nosZ* clades I and II). The *nosZ* clade I encompasses a well-known clade of nitrous oxide reductase (Henry et al., 2006), whereas clade II is a recent discovery (Sanford et al., 2012; Jones et al., 2013). As the latter occurs mostly in bacterial genomes that contain truncated versions of other denitrification genes (Graf et al., 2014), *nosZ*II harboring organisms consume rather than produce N_2_O, which has critical implications for N cycling in soils (Jones et al., 2014; Domeignoz-Horta et al., 2015). In the salt marsh chronosequence, the abundance of *nosZ*I was ca. 10-fold higher than that of clade II across all successional stages (Figure 3C). This contrasts with previous findings, where the mean relative abundance of both clades was reported to be similar (Jones et al., 2013). Moreover, the relative abundances of *nosZ*I and *nosZ*II correlated with different soil parameters (Figure 4), thus supporting the contention of niche partitioning. Jones et al. (2013) and Domeignoz-Horta et al. (2015), using structural equation modeling and correlational analyses, reported the chemical and physical properties that drove the distribution of these clades. Whereas in Jones et al. (2013) clade I was most sensitive to shifts in soil texture, shifts in soil chemistry were a more important driver of clade II. In the light of our divergent findings (Figure 4) and given the infancy of the studies on the ecology of these clades, we argue that a thorough study of the factors influencing their distributions across a range of soil biomes with contrasting biotic effects and historical contingencies is needed.

## Conclusion and methodological considerations

This study provides a resolved profile of selected genes involved in different steps of the N cycle in salt marsh soils. As mentioned, understanding the natural variations intrinsic of such communities and, in doing so, establishing a baseline genetic profile is required for future management of nitrogen dynamics in these systems (Turner et al., 2009; Deegan et al., 2012; Bowen et al., 2013). In particular, we focused on the reconstruction of community genetic potentials and specific niche partitioning of selected genes. Through this vantage point, historical contingency, niche structure and varying abiotic variables emerged as key drivers of the ecological distribution and abundance of N cycling genes along the investigated eco-evolutionary chronosequence. Of particular importance, caution should be taken in the use of gene quantities as “proxies” for the respective activities in soils, as the abundance and structure of the functional communities do not necessarily reflect their effectiveness (see Brankatschk et al., 2011; Sterngren et al., 2015). Finally, we argue that future programs aiming to monitor coastal habitats, and restore impacted ones, may profit from the methodological and scientific improvements shown herein. The current robust inventory of N cycle genes and their implementation in a successional framework in the salt marsh soils is a key achievement, and the dynamics and patterns we report can aid in the development of ecological models that aim to explain ecosystem processes and help the recovery of disturbed salt marsh ecosystems.

## Author contributions

FD-A, JDvE and JFS designed the research; JDvE and JFS contributed with new reagents and analytical tools; FD-A and MJLB performed the research and analysed the data; FD-A wrote the manuscript with comments provided by MJLB, JDvE and JFS.

### Conflict of interest statement

The authors declare that the research was conducted in the absence of any commercial or financial relationships that could be construed as a potential conflict of interest. The reviewer AV and handling Editor declared their shared affiliation, and the handling Editor states that the process nevertheless met the standards of a fair and objective review.

## Supplementary material

Supplementary Figure 1 **Map of the island of Schiermonnikoog, the Netherlands (N53°30' E6°10')**. Dot marks represent the location of the five soil successional stages established along the chronosequence in 2012 (that is, stages 0, 5, 35, 65 and 105—in years of soil development). The below panel displays photographs of each plot taken in July 2012.

Supplementary Figure 2 **Absolute quantifications of N cycling genes at five successional stages of the salt marsh chronosequence**. Data encompass four sampling times (May, July, September and November 2012). Values are shown as log copy number per gram of dry-weight (dw) soil.

Supplementary Table 1 **Location and physico-chemical parameters measured for the samples collected along the salt marsh chronosequence at the island Schiermonnikoog, The Netherlands**. Sampling was performed in the referred months in the year of 2012.

Supplementary Table 2 **Characteristics of the metagenomes generated by Illumina HiSeq2000 for soil samples collected along the salt marsh chronosequence at the island of Schiermonnikoog, The Netherlands**.

Supplementary Table 3 **List of KO IDs involved in N cycle transformations**. The genetic potential of each step was calculated as previously described (Lauro et al., 2011; Llorens-Marès et al., 2015).

Supplementary Table 4 **Overall and pairwise comparisons of KOs community profiles across five soil successional stages analyzed using PERMANOVA (Bray-Curtis distance) with 10^**3**^ permutations**. Abbreviations: MS, mean sum of squares; SS, sum of squares. ^**^*P* ≤ 0.01, ^*^*P* < 0.05.

Supplementary Table 5 **Normalized percentage (%) of annotated KOs involved in N cycle transformations across metagenomes**.

Supplementary Table 6 **Layer decision of KOs involved in N cycle transformations identified using random forest analysis (Breiman, 2001) with 1,000 trees followed by the Boruta algorithm for feature selection (average *z*-scores of 1000 runs > 4) (Kursa and Rudnicki, 2010)**.

Supplementary Table 7 **Normalized relative abundance of N cycle transformations as a proxy of the potential *in situ* relevance of each step at different soil stages along succession**.
