# Supplementary figures and images for: Reconstructing the Genetic Potential of the Microbially-Mediated Nitrogen Cycle in a Salt Marsh Ecosystem
Source: Front Microbiol. 2016 Jun 15;7:902. doi: 10.3389/fmicb.2016.00902 (PMC4908922; doi:10.3389/fmicb.2016.00902)

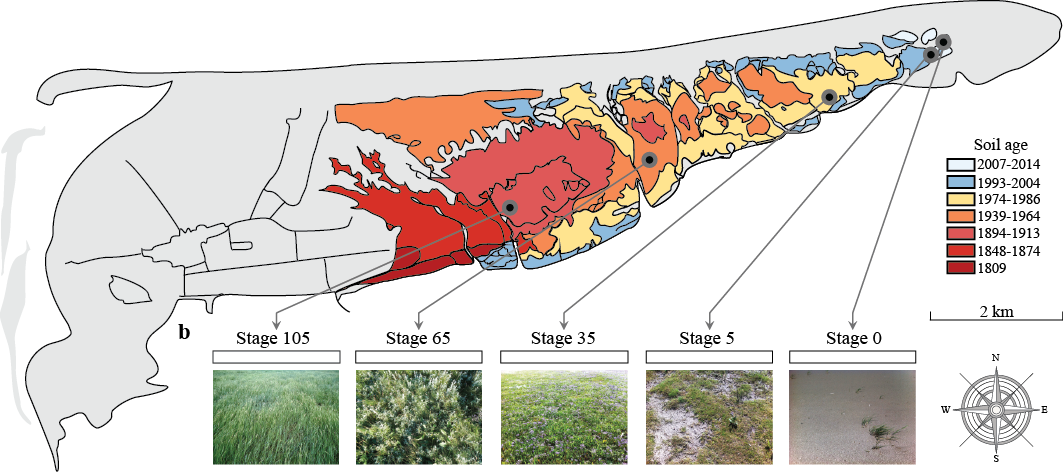

Supplement: Supplementary Figure 1 — Map of the island of Schiermonnikoog, the Netherlands (N53°30' E6°10'). Dot marks represent the location of the five soil successional stages established along the chronosequence in 2012 (that is, stages 0, 5, 35, 65 and 105—in years of soil development). The below panel displays photographs of each plot taken in July 2012. [file Image1.png]

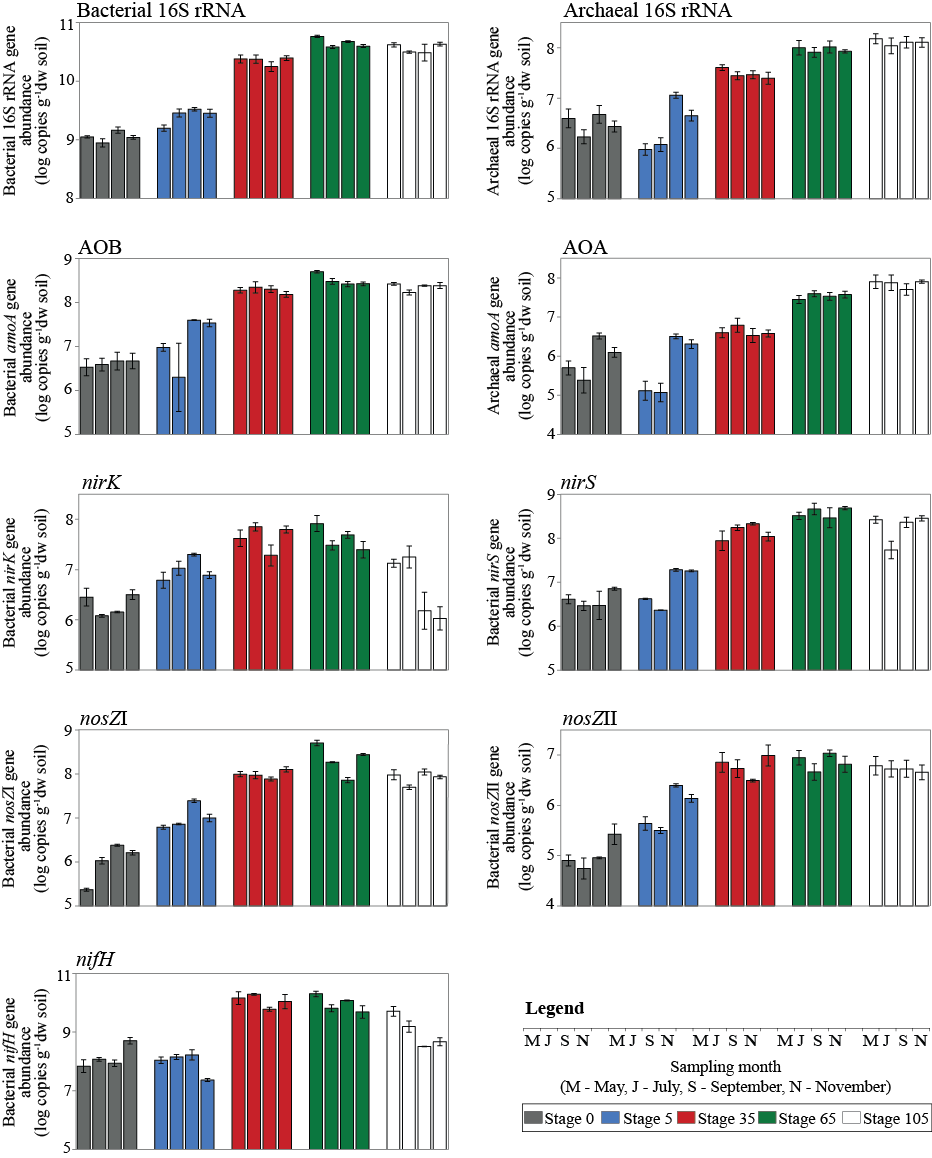

Supplement: Supplementary Figure 2 — Absolute quantifications of N cycling genes at five successional stages of the salt marsh chronosequence. Data encompass four sampling times (May, July, September and November 2012). Values are shown as log copy number per gram of dry-weight (dw) soil. [file Image2.png]
