# Supplementary material for: Reconstructing the Genetic Potential of the Microbially-Mediated Nitrogen Cycle in a Salt Marsh Ecosystem
Source: Front Microbiol. 2016 Jun 15;7:902. doi: 10.3389/fmicb.2016.00902 (PMC4908922; doi:10.3389/fmicb.2016.00902)
Supplement: Supplementary Table 1 — Location and physico-chemical parameters measured for the samples collected along the salt marsh chronosequence at the island Schiermonnikoog, The Netherlands. Sampling was performed in the referred months in the year of 2012. [file Table1.DOC]

**Supplementary Table 1.** Location and physico-chemical parameters measured for the samples collected along the salt marsh chronosequence at the island Schiermonnikoog, The Netherlands. Sampling was performed in the referred months in the year of 2012.

|  | **Stage of succession (in years)** | | | | | | | | | | | | | | | | | | | |
| --- | --- | --- | --- | --- | --- | --- | --- | --- | --- | --- | --- | --- | --- | --- | --- | --- | --- | --- | --- | --- |
|  | **Stage 0** | | | | **Stage 5** | | | | **Stage 35** | | | | **Stage 65** | | | | **Stage 105** | | | |
|  |  |  |  |  |  |  |  |  |  |  |  |  |  |  |  |  |  |  |  |  |
| **Soil physical structure** |  |  |  |  |  |  |  |  |  |  |  |  |  |  |  |  |  |  |  |  |
| Silt (%) | 2.7±0.5 | | | | 2.9±0.1 | | | | 22.3±0.4 | | | | 44.5±2.2 | | | | 49.0±0.7 | | | |
| Clay (%) | 5.0±0.0 | | | | 5.0±0.0 | | | | 23.4±1.5 | | | | 36.2±1.3 | | | | 37.7±0 | | | |
| Sand (%) | 92.3±0.5 | | | | 92.1±0.1 | | | | 54.3±1.5 | | | | 19.3±1.0 | | | | 13.3±0.7 | | | |
|  |  |  |  |  |  |  |  |  |  |  |  |  |  |  |  |  |  |  |  |  |
| **Soil chemical parameters** | **Sampling time (in the year of 2012)** | | | | | | | | | | | | | | | | | | | |
|  | **May^a^** | **July^a,b^** | **Sept^a^** | **Nov^a^** | **May^a^** | **July^a,b^** | **Sept^a^** | **Nov^a^** | **May^a^** | **July^a,b^** | **Sept^a^** | **Nov^a^** | **May^a^** | **July^a,b^** | **Sept^a^** | **Nov^a^** | **May^a^** | **July^a,b^** | **Sept^a^** | **Nov^a^** |
| Soil water content (%) | 11±1 | 11±1 | 12±1 | 13±1 | 7±1 | 7±1 | 10±1 | 13±0 | 36±2 | 38±2 | 36±3 | 38±1 | 48±1 | 46±2 | 45±3 | 56±4 | 38±2 | 35±1 | 35±1 | 44±3 |
| pH | 8.7±0.1 | 8.7±0.1 | 8.6±0.1 | 8.6±0.1 | 8.3±0.1 | 8.3±0.1 | 8.3±0.1 | 8.3±0.1 | 7.7±0.1 | 7.8±0.1 | 7.8±0.1 | 7.8±0.0 | 7.4±0.0 | 7.4±0.1 | 7.4±0.1 | 7.4±0.1 | 7.6±0.0 | 7.4±0.1 | 7.4±0.1 | 7.5±0.1 |
| Soil organic matter (g dm^-3^) | 1±0 | 1±0 | 1±0 | 1±0 | 2±1 | 2±1 | 2±0 | 3±1 | 17±1 | 15±1 | 14±1 | 15±1 | 47±1 | 38±2 | 33±1 | 33±2 | 32±2 | 31±1 | 34±2 | 28±1 |
| Sulphate (mg dm^-3^) | 16±3 | 13±1 | 95±4 | 75±4 | 32±4 | 43±5 | 180±4 | 203±9 | 670±13 | 130±12 | 113±10 | 53±4 | 704±5 | 550±26 | 452±23 | 67±11 | 539±13 | 423±12 | 498±5 | 416±26 |
| Sodium (mg dm^-3^) | 155±13 | 178±19 | 752±88 | 647±182 | 170±15 | 187±9 | 1248±38 | 1587±89 | 6836±240 | 3782±340 | 4799±457 | 3755±94 | 4533±384 | 6447±204 | 5888±346 | 5200±438 | 5188±624 | 3541±170 | 4654±731 | 5039±687 |
| Total nitrogen (mg kg^-1^) | 462±50 | 329±44 | 371±116 | 378±105 | 490±76 | 530±74 | 467±172 | 478±78 | 3232±150 | 3253±389 | 2606±539 | 2679±209 | 9604±1187 | 8132±1955 | 8580±1785 | 8276±1212 | 6300±1646 | 5833±3003 | 5693±2660 | 5087±2151 |
| Ammonium (mg kg^-1^) | 5±1 | 4±1 | 4±1 | 4±1 | 6±1 | 6±1 | 5±2 | 6±1 | 38±3 | 38±4 | 30±6 | 33±4 | 115±16 | 94±18 | 102±21 | 99±15 | 75±20 | 70±37 | 67±32 | 60±30 |
| Nitrate (mg kg^-1^) | 3±0 | 2±0 | 2±0 | 2±0 | 3±1 | 3±1 | 3±1 | 3±1 | 21±2 | 21±3 | 17±4 | 18±2 | 63±7 | 51±8 | 57±12 | 55±8 | 42±11 | 39±20 | 38±18 | 34±17 |

^a^qPCR assays

^b^Shotgun metagenome sequencing
