# Supplementary material for: Reconstructing the Genetic Potential of the Microbially-Mediated Nitrogen Cycle in a Salt Marsh Ecosystem
Source: Front Microbiol. 2016 Jun 15;7:902. doi: 10.3389/fmicb.2016.00902 (PMC4908922; doi:10.3389/fmicb.2016.00902)
Supplement: Supplementary Table 2 — Characteristics of the metagenomes generated by Illumina HiSeq2000 for soil samples collected along the salt marsh chronosequence at the island of Schiermonnikoog, The Netherlands. [file Table2.DOC]

**Supplementary Table 2.** Characteristics of the metagenomes generated by Illumina HiSeq2000 for soil samples collected along the salt marsh chronosequence at the island of Schiermonnikoog, The Netherlands.

|  | **Stage of succession (in years)** | | | | | | | | | | | | | | |
| --- | --- | --- | --- | --- | --- | --- | --- | --- | --- | --- | --- | --- | --- | --- | --- |
|  | **Stage 0** | | | **Stage 5** | | | **Stage 35** | | | **Stage 65** | | | **Stage 105** | | |
|  | **Plot A** | **Plot B** | **Plot C** | **Plot A** | **Plot B** | **Plot C** | **Plot A** | **Plot B** | **Plot C** | **Plot A** | **Plot B** | **Plot C** | **Plot A** | **Plot B** | **Plot C** |
| **Geographic coordinates** |  |  |  |  |  |  |  |  |  |  |  |  |  |  |  |
| Latitude | 53°30’18’’N | 53°30’18’’N | 53°30’18’’N | 53°30’7’’N | 53°30’7’’N | 53°30’7’’N | 53°29’59’’N | 53°29’59’’N | 53°29’59’’N | 53°29’35’’N | 53°29’35’’N | 53°29’35’’N | 53°28’56’’N | 53°28’56’’N | 53°28’56’’N |
| Longitude | 6°19’51’’E | 6°19’51’’E | 6°19’51’’E | 6°19’54’’E | 6°19’54’’E | 6°19’54’’E | 6°18’57’’E | 6°18’57’’E | 6°18’57’’E | 6°16’20’’E | 6°16’20’’E | 6°16’20’’E | 6°14’3’’E | 6°14’3’’E | 6°14’3’’E |
| ***Metagenomes** |  |  |  |  |  |  |  |  |  |  |  |  |  |  |  |
| MG-RAST ID | 4558897.3 | 4558900.3 | 4558903.3 | 4558906.3 | 4558909.3 | 4558912.3 | 4558915.3 | 4558918.3 | 4558921.3 | 4558924.3 | 4558927.3 | 4558930.3 | 4558933.3 | 4558936.3 | 4558939.3 |
| Metagenome size (bp) | 1 946 098 904 | 3 039 009 260 | 1 729 121 135 | 1 985 828 415 | 2 470 040 059 | 2 541 714 674 | 2 876 930 138 | 1 658 957 366 | 2 169 497 340 | 1 883 521 083 | 1 814 228 985 | 1 103 890 408 | 384 386 953 | 503 545 455 | 2 659 532 084 |
| Average sequence length (bp) | 156 ± 27 | 157 ± 28 | 159 ± 29 | 164 ± 32 | 159 ± 29 | 150 ± 24 | 156 ± 26 | 157 ± 28 | 155 ± 27 | 155 ± 27 | 165 ± 33 | 167 ± 32 | 170 ± 34 | 167 ± 34 | 156 ± 28 |
| Number of sequences | 12 450 878 | 19 321 585 | 10 839 811 | 12 107 696 | 15 534 513 | 16 934 258 | 18 408 001 | 10 533 784 | 13 947 451 | 12 123 756 | 10 969 050 | 6 599 703 | 2 258 688 | 2 999 891 | 16 987 073 |
| GC content (%) | 56 ± 12 | 56 ± 12 | 54 ± 12 | 56 ± 12 | 56 ± 12 | 56 ± 12 | 55 ± 11 | 54 ± 11 | 55 ± 11 | 57 ± 10 | 56 ± 11 | 55 ± 11 | 55 ± 11 | 57 ± 10 | 57 ± 10 |
| Number of predicted ORFs | 11 596 311 | 17 647 296 | 10 070 537 | 11 124 734 | 14 473 485 | 15 374 314 | 17 155 698 | 9 823 968 | 13 024 803 | 11 275 236 | 10 201 327 | 6 140 974 | 2 057 545 | 2 794 317 | 15 889 995 |
| ORFs with predicted function | 3 934 757 | 5 853 920 | 3 272 425 | 4 105 274 | 5 645 080 | 5 925 433 | 5 285 542 | 3 104 792 | 3 894 995 | 3 794 182 | 3 610 530 | 2 181 005 | 755 878 | 1 025 318 | 5 627 796 |
| Bacteria (%) | 96.1 | 95.8 | 95.7 | 97 | 97.5 | 97.5 | 95.5 | 95.6 | 95.8 | 96.7 | 97.2 | 96.9 | 97.1 | 97.1 | 97.1 |
| Eukarya (%) | 2.1 | 2.5 | 2.4 | 1.8 | 1.6 | 1.8 | 2.1 | 2.1 | 1.9 | 1.5 | 1.4 | 1.5 | 1.8 | 1.3 | 1.3 |
| Archaea (%) | 1.5 | 1.4 | 1.6 | 1.1 | 0.8 | 0.6 | 2.1 | 2 | 2.1 | 1.6 | 1.2 | 1.4 | 0.9 | 1.4 | 1.4 |
| KO matches | 1 565 010 | 2 391 486 | 1 293 459 | 1 618 989 | 2 173 227 | 2 387 195 | 2 065 112 | 1 202 620 | 1 526 363 | 1 486 222 | 1 392 578 | 835 141 | 298 105 | 396 573 | 2 188 639 |

^*^Post QC values
