# Supplementary material for: Reconstructing the Genetic Potential of the Microbially-Mediated Nitrogen Cycle in a Salt Marsh Ecosystem
Source: Front Microbiol. 2016 Jun 15;7:902. doi: 10.3389/fmicb.2016.00902 (PMC4908922; doi:10.3389/fmicb.2016.00902)
Supplement: Supplementary Table 3 — List of KO IDs involved in N cycle transformations. The genetic potential of each step was calculated as previously described (Lauro et al., 2011; Llorens-Marès et al., 2015). [file Table3.DOC]

**Supplementary Table 3.** List of KO IDs involved in N cycle transformations. The genetic potential of each step was calculated as previously described (Lauro et al., 2011; Llorens-Marès et al., 2015).

| **N cycle transformation** | **KO ID** | **Marker gene** | **Genetic potential** |
| --- | --- | --- | --- |
| **Ammonification** | K03385 | formate-dependent nitrite reductase periplasmic cytochrome c552 (*nrfA*) | K03385 |
| **Anammox** | K07174 | hydrazine oxidoreducatse (*hzo*)* | K07174 |
| **Denitrification** | K00376 | nitrous oxide reductase (*nosZ*) | (K00376+K02305+  K04561+)/3 |
|  | K02305 | nitric-oxide reductase (*norC*) |  |
|  | K04561 | nitric-oxide reductase (*norB*) |  |
| **Nitrate reduction + Nitrite oxidation** | K00370 | nitrate reductase alpha & nitrite oxidoreductase (*narG/nxrA*) | (K00370+K00371)/2 |
|  | K00371 | nitrate reductase beta & nitrite oxidoreductase (*narH/nxrB*) |  |
| **Nitrate reduction** | K02567 | periplasmic nitrate reductase (*napA*) | (K02567+K02568)/2 |
|  | K02568 | cytochrome c-type protein (*napB*) |  |
| **Nitrification** | K10944 | ammonia monooxygenase subunit A (*amoA*) | (K10944+K10945+  K10946)/3 |
|  | K10945 | ammonia monooxygenase subunit B (*amoB*) |  |
|  | K10946 | ammonia monooxygenase subunit C (*amoC*) |  |
| **Nitrogen assimilation** | K00360 | assimilatory nitrate reductase | (K00360+K00367+  K01915+K00265+  K00284)/3 |
|  | K00367 | assimilatory nitrate reductase |  |
|  | K01915 | glutamine synthetase (*glnA*) |  |
|  | K00265 | glutamate synthase (NADPH/NADH) large chain (*gltB*) |  |
|  | K00284 | glutamate synthase (ferredoxin-dependent) (*gltS*) |  |
| **Nitrogen Fixation** | K00531 | nitrogenase* | (K00531+K02586+  K02588+K02591)/4 |
|  | K02586 | nitrogenase molybdenum-iron protein alpha chain (*nifD*) |  |
|  | K02588 | nitrogenase iron protein (*nifH*) |  |
|  | K02591 | nitrogenase molybdenum-iron protein beta chain (*nifK*) |  |
| **Nitrogen Mineralization** | K00260 | glutamate dehydrogenase | K00260+K00261+  K00262 |
|  | K00261 | glutamate dehydrogenase |  |
|  | K00262 | glutamate dehydrogenase |  |

^*^not found
