# Supplementary material for: Reconstructing the Genetic Potential of the Microbially-Mediated Nitrogen Cycle in a Salt Marsh Ecosystem
Source: Front Microbiol. 2016 Jun 15;7:902. doi: 10.3389/fmicb.2016.00902 (PMC4908922; doi:10.3389/fmicb.2016.00902)
Supplement: Supplementary Table 4 — Overall and pairwise comparisons of KOs community profiles across five soil successional stages analyzed using PERMANOVA (Bray-Curtis distance) with 103 permutations. Abbreviations: MS, mean sum of squares; SS, sum of squares. **P ≤ 0.01, *P < 0.05. [file Table4.DOC]

**Supplementary Table 4.** Overall and pairwise comparisons of KOs community profiles across five soil successional stages analyzed using PERMANOVA (Bray-Curtis distance) with 10^3^ permutations. Abbreviations: MS, mean sum of squares; SS, sum of squares. ***P*≤0.01, **P*<0.05.

| **KEEG Orthology (KOs)** | | | | | |
| --- | --- | --- | --- | --- | --- |
| **Source** | **d.f.** | **SS** | **MS** | **Pseudo-F** | **P-value** |
| Successional stages | 4 | 282.24 | 70.559 | 6.7683 | 0.001** |
| Residuals | 10 | 104.25 | 10.425 |  |  |
| Total | 14 | 386.48 |  |  |  |
|  |  |  |  |  |  |
| **Average similarity between stages** | | |  |  |  |
|  | **Stage 0** | **Stage 5** | **Stage 35** | **Stage 65** | **Stage 105** |
| Stage 0 | 96.267 |  |  |  |  |
| Stage 5 | 92.361 | 95.262 |  |  |  |
| Stage 35 | 92.801 | 91.858 | 96.612 |  |  |
| Stage 65 | 91.003 | 92.525 | 93.597 | 95.363 |  |
| Stage 105 | 90.310 | 92.254 | 92.086 | 94.408 | 94.222 |
| **Pairwise tests between consecutive successional stages** | | | |  |  |
|  | **t** | **P-value** |  |  |  |
| Stages 0 to 5 | 2.780 | 0.009** |  |  |  |
| Stages 5 to 35 | 3.128 | 0.01** |  |  |  |
| Stages 35 to 65 | 2.336 | 0.016* |  |  |  |
| Stages 65 to 105 | 1.235 | 0.278 |  |  |  |
| **Selected KOs for N cycle** | | | | | |
| **Source** | **d.f.** | **SS** | **MS** | **Pseudo-F** | **P-value** |
| Successional stages | 4 | 720.86 | 180.21 | 7.7958 | 0.001** |
| Residuals | 10 | 231.17 | 23.117 |  |  |
| Total | 14 | 952.03 |  |  |  |
| **Average similarity between stages** | | |  |  |  |
|  | **Stage 0** | **Stage 5** | **Stage 35** | **Stage 65** | **Stage 105** |
| Stage 0 | 94.572 |  |  |  |  |
| Stage 5 | 88.368 | 91.396 |  |  |  |
| Stage 35 | 89.388 | 81.251 | 96.208 |  |  |
| Stage 65 | 90.651 | 87.788 | 89.176 | 94.286 |  |
| Stage 105 | 89.749 | 89.895 | 85.133 | 91.542 | 91.532 |
|  |  |  |  |  |  |
| **Pairwise tests between consecutive successional stages** | | | |  |  |
|  | **t** | **P-value** |  |  |  |
| Stages 0 to 5 | 2.467 | 0.024* |  |  |  |
| Stages 5 to 35 | 4.608 | 0.005** |  |  |  |
| Stages 35 to 65 | 3.586 | 0.005** |  |  |  |
| Stages 65 to 105 | 1.539 | 0.109 |  |  |  |
