# Supplementary material for: Reconstructing the Genetic Potential of the Microbially-Mediated Nitrogen Cycle in a Salt Marsh Ecosystem
Source: Front Microbiol. 2016 Jun 15;7:902. doi: 10.3389/fmicb.2016.00902 (PMC4908922; doi:10.3389/fmicb.2016.00902)
Supplement: Supplementary Table 5 — Normalized percentage (%) of annotated KOs involved in N cycle transformations across metagenomes. [file Table5.DOC]

**Supplementary Table 5.** Normalized percentage (%) of annotated KOs involved in N cycle transformations across metagenomes.

|  | **Stage 0** | **Stage 5** | **Stage 35** | **Stage 65** | **Stage 105** |
| --- | --- | --- | --- | --- | --- |
| **Ammonification** | 1.0 | 0.6 | 0.6 | 0.7 | 0.5 |
| **Denitrification** | 5.8 | 4.3 | 6.3 | 4.7 | 5.0 |
| **Nitrate reduction + Nitrite oxidation** | 6.7 | 5.0 | 6.0 | 6.0 | 6.0 |
| **Nitrate reduction** | 3.2 | 2.3 | 4.4 | 2.3 | 2.0 |
| **Nitrification** | 0.2 | 0.3 | 0.1 | 0.2 | 0.2 |
| **Nitrogen assimilation** | 58.8 | 64.4 | 57.4 | 64.6 | 63.7 |
| **Nitrogen fixation** | 0.6 | 0.2 | 2.4 | 1.4 | 0.6 |
| **Nitrogen mineralization** | 23.7 | 22.8 | 22.9 | 20.2 | 22.1 |
