# Supplementary material for: Reconstructing the Genetic Potential of the Microbially-Mediated Nitrogen Cycle in a Salt Marsh Ecosystem
Source: Front Microbiol. 2016 Jun 15;7:902. doi: 10.3389/fmicb.2016.00902 (PMC4908922; doi:10.3389/fmicb.2016.00902)
Supplement: Supplementary Table 6 — Layer decision of KOs involved in N cycle transformations identified using random forest analysis (Breiman, 2001) with 1,000 trees followed by the Boruta algorithm for feature selection (average z-scores of 1000 runs > 4) (Kursa and Rudnicki, 2010). [file Table6.DOC]

**Supplementary Table 6.** Layer decision of KOs involved in N cycle transformations identified using random forest analysis (Breiman, 2001) with 1,000 trees followed by the Boruta algorithm for feature selection (average *z-*scores of 1000 runs > 4) (Kursa and Rudnicki, 2010).

| **KO ID** | **Layer decision** | **Layer importance** |
| --- | --- | --- |
| K00260 | ***Confirmed*** | 5.7249 |
| K00261 | ***Confirmed*** | 4.8351 |
| K00262 | ***Confirmed*** | 4.7245 |
| K00265 | ***Confirmed*** | 4.6379 |
| K00284 | Rejected | Inf |
| K00360 | ***Confirmed*** | 4.8668 |
| K00367 | Rejected | Inf |
| K00370 | Rejected | Inf |
| K00371 | Rejected | Inf |
| K00376 | Rejected | Inf |
| K01915 | ***Confirmed*** | 4.1363 |
| K02305 | Rejected | Inf |
| K02567 | ***Confirmed*** | 4.9072 |
| K02568 | ***Confirmed*** | 4.8981 |
| K02586 | ***Confirmed*** | 7.5560 |
| K02588 | ***Confirmed*** | 5.7977 |
| K02591 | ***Confirmed*** | 7.0467 |
| K03385 | ***Confirmed*** | 4.9127 |
| K04561 | ***Confirmed*** | 6.0213 |
| K10944 | Rejected | Inf |
| K10945 | Rejected | Inf |
| K10946 | Rejected | Inf |
