# Supplementary material for: Reconstructing the Genetic Potential of the Microbially-Mediated Nitrogen Cycle in a Salt Marsh Ecosystem
Source: Front Microbiol. 2016 Jun 15;7:902. doi: 10.3389/fmicb.2016.00902 (PMC4908922; doi:10.3389/fmicb.2016.00902)
Supplement: Supplementary Table 7 — Normalized relative abundance of N cycle transformations as a proxy of the potential in situ relevance of each step at different soil stages along succession. [file Table7.DOC]

**Supplementary Table 7.** Normalized relative abundance of N cycle transformations as a proxy of the potential *in situ* relevance of each step at different soil stages along succession.

|  | **Stage 0 (%)** | **Stage 5 (%)** | **Stage 35 (%)** | **Stage 65 (%)** | **Stage 105 (%)** |
| --- | --- | --- | --- | --- | --- |
| **Ammonification** | 2.0 | 1.3 | 1.2 | 1.4 | 1.1 |
| **Denitrification** | 3.7 | 2.9 | 4.1 | 3.2 | 3.4 |
| **Nitrate reduction + Nitrite oxidation** | 6.5 | 5.0 | 5.9 | 6.1 | 6.1 |
| **Nitrate reduction** | 3.2 | 2.2 | 4.4 | 2.4 | 2.0 |
| **Nitrification** | 0.1 | 0.2 | 0.1 | 0.1 | 0.1 |
| **Nitrogen assimilation** | 38.1 | 42.8 | 37.7 | 44.3 | 42.7 |
| **Nitrogen fixation** | 0.4 | 0.2 | 1.6 | 1.0 | 0.4 |
| **Nitrogen mineralization** | 46.0 | 45.4 | 45.1 | 41.5 | 44.4 |
